# Significant biomarkers for predicting 1-month changes in IGF-1 in growth hormone-deficient children following r-hGH therapy: Biomarkers predicting changes in IGF-1 in GHD children after r-hGH therapy

**DOI:** 10.3724/abbs.2024089

**Published:** 2024-06-06

**Authors:** Fei Liu, NokI Lei, Wunying Li, Yiwen Zheng, Liangjian Hu, Ronggui Hu, Wenli Lu, Yu S. Huang

**Affiliations:** 1 Drug Discovery and Design Center State Key Laboratory of Drug Research Shanghai Institute of Materia Medical Chinese Academy of Sciences Shanghai 201203 China; 2 Department of Pediatrics Ruijin Hospital Affiliated to Shanghai Jiao Tong University Shanghai 230025 China; 3 University of Chinese Academy of Sciences Beijing 100049 China; 4 Department of Statistics Donghua University Shanghai 201620 China; 5 State Key Laboratory of Molecular Biology Shanghai Institute of Biochemistry and Cell Biology University of Chinese Academy of Sciences Shanghai 200031 China; 6 Genecast Corp. Ltd. Beijing 100089 China

Growth hormone deficiency (GHD) is the most common pituitary hormone deficiency and is clinically characterized by short stature, delayed bone age and central distribution of body fat, and it has also been proven to be mildly heritable. Treatment with recombinant human growth hormone (r-hGH) is primary and safe for GHD children, and a dose of 0.15‒0.20 mg/kg each week results in a considerable increase in height velocity, with noteworthy growth during the first year of therapy [1]. Previous studies have shown that serum IGF-1 is strongly correlated with the growth response [2]. Therefore, IGF-1 can serve as a clinical indicator for monitoring compliance, efficacy and safety. However, the response to GH therapy shows significant individual variation, which is strongly associated with genetic factors. The prevalence rate of severe childhood GHD-related short stature varies from 1:4000 to 1:10,000 [3], while approximately 3%‒4% of the population in China suffers from short stature with an increasing trend. Therefore, an open-label, prospective, multicentric, noncomparative, nonrandomized phase IV interventional study (NCT01187550, Merck Serono Study 27709) was conducted to investigate the relationship between the prospective biomarkers of GHD patients and the individual variation in the primary therapeutic response following 4 weeks of r-hGH therapy.

Given the significance of predicting GHD treatment response and the gaps in previous research, we sought to adopt a comprehensive strategy to accurately predict the therapeutic response utilizing the transcriptome, single nucleotide polymorphisms (SNPs) and clinical factors. We employed continuous variables and standard deviation scores of differences in serum IGF-1 levels after 4 weeks of r-hGH therapy (ΔIGF-1) as targets to filter possible influencing variables. Furthermore, we compared several potential machine learning techniques, validated by PCA and PLS-DA, and ultimately applied the elastic net algorithm to determine the optimized predictive factors with consistent effect sizes. Additionally, expression quantitative trait locus (eQTL) analysis and differentially expressed gene (DEG) analysis were conducted to identify significant biomarkers for GHD treatment.

A total of 204 GHD prepubertal subjects were recruited from 9 medical centers for the clinical trial NCT01187550 in China. For the purpose of identifying the appropriate potential predictable genetic factors, we excluded patients with the confounding factor of age, as the distribution of ΔIGF-1 varied significantly at age 16 ( Supplementary Figure S1). After the removal of missing values, 132 patients under the age of 16 ( Table 1) were enrolled. To correct the effect size of potential influencing genetic factors, we investigated the nonlinear relationships between all clinical variables via t-SNE ( Figure 1A) and explored the linear relationships between the baseline clinical variables and ΔIGF-1 via a correlation heatmap ( Figure 1B), which indicated that BMI, baseline IGF-1, baseline GH peak and baseline IGFBP3 were significantly associated with ΔIGF-1 ( *P*<0.05). Further removing redundancy with the 4 individually significant clinical variables via multivariate linear regression, only BMI and baseline IGF-1 were found to be potential predictors of treatment response ( *P*<0.01; Supplementary Table S1), as shown in Figure 1A,B. The impact of sex and gestational age were not considered in this study since neither of these factors was significantly different ( Supplementary Figure S2). To identify significant associations between SNPs and treatment response, a total of 1,536 SNPs were genotyped on a custom-designed Illumina GoldenGate microarray and selected from three GHD-related pathways: 1) the GH-IGF-1 pathway axis; and 2) bone and cell growth; and 3) glucose and lipid metabolism ( Supplementary Table S2). A total of 1202 SNPs satisfying the following three criteria were retained for further analyses of Plink: 1) missing value <5%; 2) Hardy-Weinberg equilibrium test *P*>0.001; and 3) minor allele frequency (MAF)>0.05. Thirty-eight SNPs corresponding to 18 genes were significantly correlated with ΔIGF-1 ( *P*<0.05; Figure 1C and Supplementary Table S3), which is mostly related to cell growth and the IGF-1 pathway ( Supplementary Table S4), and 4 SNPs belonging to the *HSD3B1* and *INSR* genes were significantly correlated ( *P*<0.01; Figure 1D and Supplementary Table S5). *GRB10*, *IGF1R*, *SLC2A4*, and *PTPN1* are strongly associated with the growth hormone downstream response and were identified in previous studies [4]. *INSR* is significantly associated with the GH-IGF-1 axis and is involved in the MAPK/ERK and PI3K/AKT pathways [5]. *IGF2*, *CDKs*, *FGFR1*, *FGFR2*, *AKT3*, *TGFB3* and *CREM* were found to be specific genes related to the response to GHD therapy in the final prediction model. Cell growth and bone formation have been demonstrated to be impacted by FGFR1 and FGFR2 [6], while the treatment response for GHD has been shown to be influenced by CREM [7]. The partial consistency with prior research makes this prediction model more accurate, and the discovery of novel genes with significant effects demonstrates that the complex trait of height is, in fact, controlled by several genetic variables and offers new research directions.

**Figure 1:**
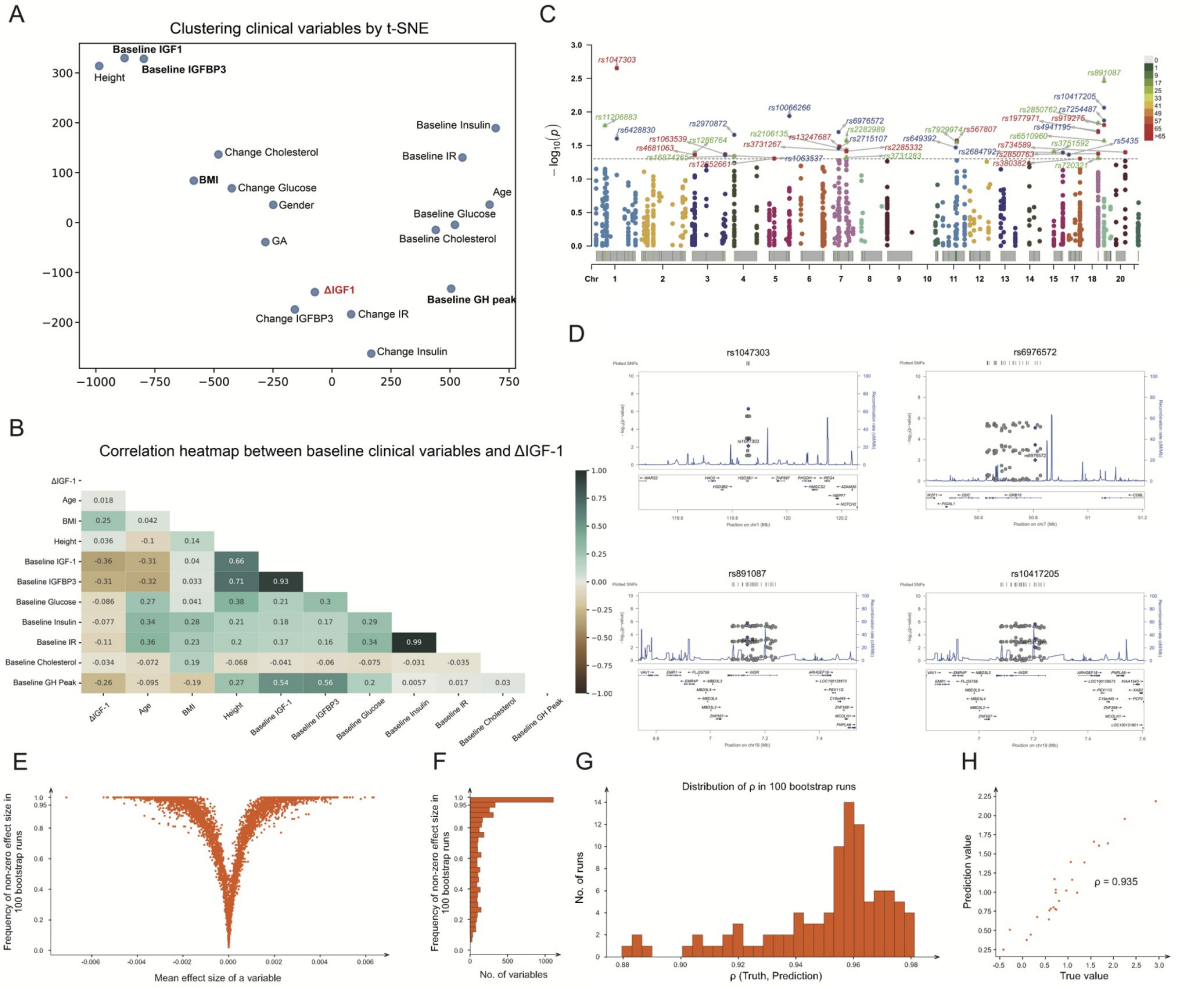
Figure 1 Identification of predictive factors for ΔIGF-1 through correlation, association and modelling analyses 1. Correlations between clinical variables and ΔIGF-1. (A) Clustering of all clinical variables by t-SNE with perplexity=3. ΔIGF-1 is colored in red. Four clinical variables, BMI, baseline IGF-1, baseline GH peak, and baseline IGFBP3, which were significantly associated with ΔIGF-1 according to univariate regression, are in bold. (B) Correlation heatmap between baseline clinical variables and ΔIGF-1. BMI, baseline IGF-1, baseline IGFBP3, and baseline GH peak were the four variables most strongly correlated with ΔIGF-1. BMI was positively correlated (green), while baseline IGF-1, IGFBP3, and GH peak were negatively correlated (brown). Two pairs, baseline IGF-1 (baseline IGFBP3) and baseline IR (baseline insulin), showed a strong correlation, which explains why baseline IGFBP3 was rendered entirely redundant by baseline IGF-1. The baseline GH peak was moderately correlated (r2≈0.55) with both the baseline IGF-1 and baseline IGFBP3 levels, which was not significant according to multivariate regression. 2. Significant SNPs correlated with ΔIGF-1 corrected by covariates. (C) Manhattan plot of 40 SNPs (*P<0.05). (D) LocusZoom plots of 4 SNPs (**P<0.01). The purple rhombuses represent significant SNPs, and the purple circles are two covariates. 3. Variable selection for the final prediction model by bootstrapping. (E) Each dot represents a variable. The x-axis is the mean effect size of a variable, and the y-axis is the frequency of nonzero effect sizes in 100 bootstrap runs. The larger the mean effect size is, the more likely the variable will be selected. (F) The x-axis is the number of variables. The y-axis is the frequency of nonzero effect sizes in 100 bootstrap runs. Most variables (the longest bar with frequency=1.0) are consistently selected, which shows model consistency. (G) The distribution of ρ (Spearman’s rank correlation) between the true and predicted values for 100 bootstrap runs; the median value of this ρ is 0.957. (H) The prediction results obtained by the final prediction model for one set of validation subjects (not included in the model training) achieved a ρ of 0.935.

**Table:** **Table 1** Characteristics of the 132 subjects included in this study

	Mean	Standard deviation	Min/Max
Gender	103 male/29 female
Gestational age (month)	112 AGA/20 SGA
Age (year)	9.11	3.14	2.42/15.5
Height SDS	–3.31	1.51	–9.05/1.25
Baseline IGF-1 SDS	–1.76	1.98	–7.81/1.28
Change IGF-1 (ΔIGF-1)	1.08	1.08	–2.94/4.81
Baseline IGFBP3 SDS	–0.90	2.04	–6.97/1.91
Change IGFBP3 SDS	0.66	1.16	–2.02/5.34
Baseline S glucose	4.78	0.63	1.40/6.30
Change S glucose	0.16	0.66	–2.40/2.50
Baseline S insulin	35.77	84.88	14.0/945.0
Change S insulin	5.37	88.86	–893.0/203.0
Baseline IR	7.84	17.80	2.30/193.20
Change IR	1.63	19.37	–182.80/57.71
Baseline S cholesterol	4.16	0.77	2.15/6.39
Change S cholesterol	–0.23	0.56	–1.81/2.05
Baseline GH peak	4.73	3.06	0.04/9.90
BMI SDS	–0.28	0.94	–2.71/3.01

Abbreviations: BMI, body mass index; GHD, growth hormone deficiency; IGF-I, insulin-like growth factor-I; IGFBP3, IGF-binding protein 3; SDS, standard deviation score. ΔIGF-1 is the SDS of the difference in serum IGF-1 levels, which is the primary treatment response measure. Min and Max are the minimum and maximum values of the variable, respectively.

A total of 204 GHD prepubertal subjects were recruited in China for the clinical trial NCT01187550. Demographic data, medical history, Tanner stage, physical examination, body weight, height, bone age measurement, body mass index, review of baseline medications and procedures, and blood sampling were performed at the baseline visit, end of treatment visit (week 4) and week 4 follow-up visit. After excluding the subjects with missing values, a total of 132 prepubertal subjects under the age of 16 were recruited.

To identify potential predictive factors, we adopted the elastic net to filter genetic factors, which consisted of transcript probes and significant SNPs corrected by covariables after comparing a variety of machine learning methods ( Supplementary Figure S3). A total of 54,675 expression probes were pruned down to 4768 informative expression probes through three filters before applying the elastic net to select the following: 1) remaining gene expression probes with a standard deviation>0.25; 2) remaining gene expression probes with an absolute correlation with ΔIGF-1>0.1; and 3) pruning probes with an expression>10 in less than 85% of the subjects. To explore the optimal prediction model, a grid search algorithm with tenfold cross-validation was applied. Overall, 1217 expression probes and 2 clinical variables (with over 95% nonzero effect size in 100 bootstraps, Figure 1E,F were found to be effective at predicting treatment response. The model achieved a median ρ (Spearman’s rank correlation coefficient) of 0.957 ( Figure 1G) in 100 bootstrap runs, and Figure 1H shows that the performance of the final prediction model was verified in the independent validation set of subjects who were not involved in the model training. As shown in Supplementary Table S6, the results of the top 50 predictive probes achieved the greatest absolute effect size, and the heatmap shows that these probes and the 2 clinical variables could effectively cluster the subjects with high and low responses ( Figure 2A). PLS-DA plots were generated to demonstrate the ability of all the selected probes to classify response groups ( Figure 2B). Further evaluation by GWAS enrichment analysis of the gene transcripts included in the final model revealed that body height, blood protein measurement, and heel bone mineral density were the three most highly enriched phenotypes ( Supplementary Table S7), which validated the effectiveness of our prediction model. In particular, height was shown to be highly correlated with both baseline IGF-1 and baseline IGFBP3, highlighting the role of genetics in determining human height [ 2, 8]. The association between height and blood glucose level was further supported by a moderate correlation between height and baseline glucose (r ^2^=0.38), which is impacted by both hereditary variables impacting nutritional digestion and environmental factors, including the availability of dietary proteins [9]. Additionally, the prediction model heavily relies on genes associated with blood protein measurement and erythrocyte count, which serve as a stand-in for nutrient digestion and oxygen delivery.

**Figure 2:**
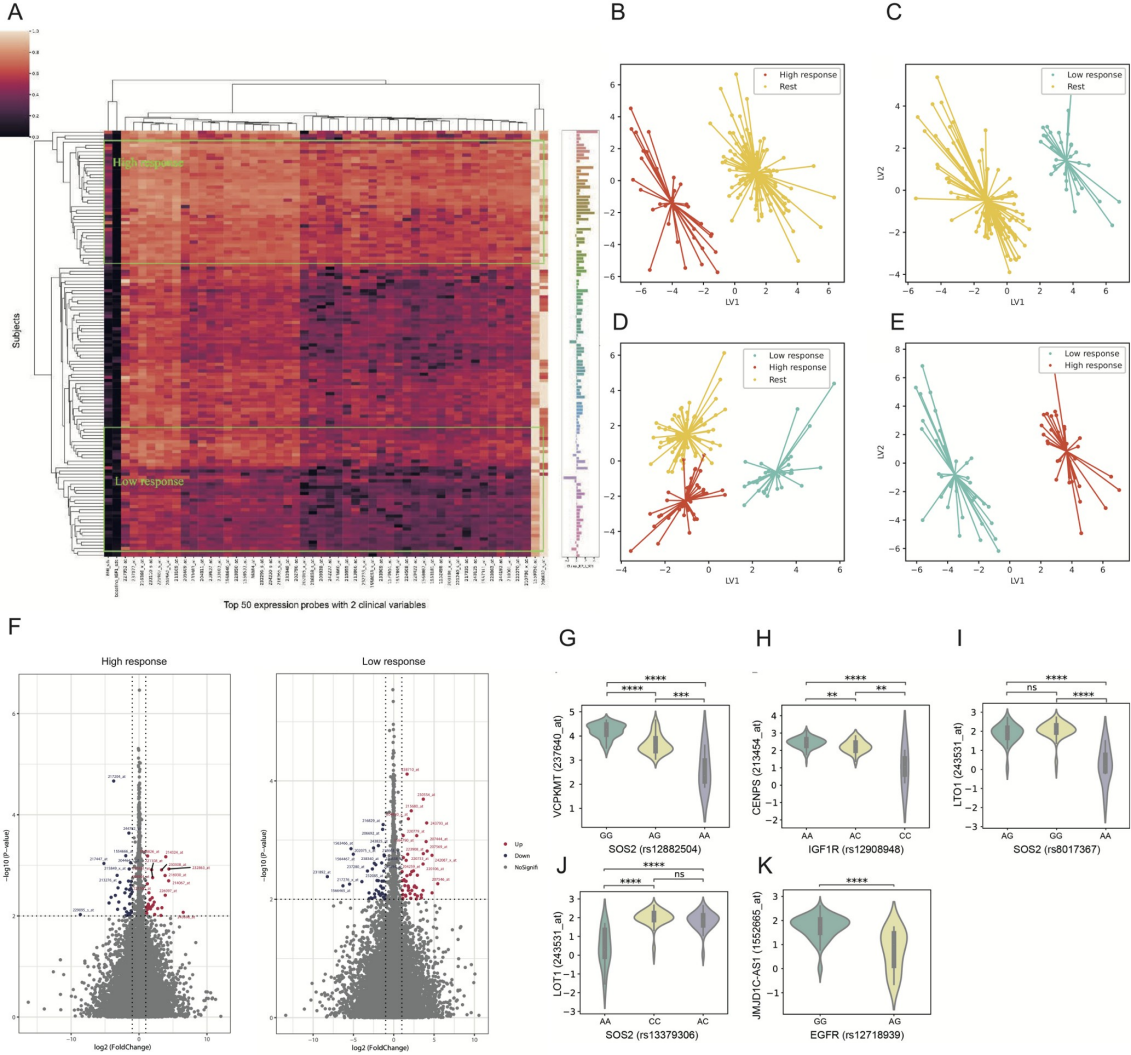
Figure 2 Identification of potential biomarkers and pathways associated with GHD treatment (A) Clustering heatmap showing that the top 50 predictive probes (Supplementary Table S6) and 2 clinical variables can be used to distinguish between high-response and low-response subjects. High response: ΔIGF-1≥Q3; low response: ΔIGF-1≤Q1. (B‒E) Partial least squares-discriminant analysis (PLS-DA) plot demonstrating the applicability of using all probes in prediction models, showing very clear clustering of GHD patients’ r-hGH therapy response regarding the different levels of ΔIGF-1. (F) Volcano plot for differential gene expression probes in different response groups. Scattered points represent expression probes: the x-axis is the log2-fold change in the ratio of r-hGH-treated probes of patients vs baseline patients, whereas the y-axis is the ‒log10 P value, indicating the probability that a probe has statistical significance in its differential expression. The red dots represent probes that were significantly overexpressed after treatment, and the blue dots represent probes that were significantly downregulated after treatment. The probes with significant fold changes in the high-response group showed a strong association with the GH-IGF-1 axis, indicating that these probes potentially promote the GHD treatment response. (G‒K) Violin plots showing the associations between SNP genotypes and expression probes (FDR<0.05). The x-axis is the SNP genotype. The y-axis is the distribution of the expression values of the expression probes. The most significant eQTL association was between SNP rs12882504 in the intronic region of SOS2 and the ex-pression probe 237640_at of VCPKMT, which are relatively close in position on the chromosome, indicating that VCPKMT is a possible gene that affects the GHD response and that SOS2 may have a regulatory function. ns: not significant; **P<0.01.

To explore whether the “body height” phenotype analyzed by GWAS is consistent with the functions of IGF-1, a KEGG pathway enrichment analysis of 16 pathways was performed by using 129 genes, which was highly consistent with the functions of IGF-1 ( Supplementary Table S8). Differentially expressed genes were identified by a significant correlation with ΔIGF-1 ( *P*<0.05). In addition, an absolute fold change>1 of each expression probe with a paired *t* test ( *P*<0.01) was used to investigate the up-/downregulated genes in the high-response group (response>Q3) and low-response group (response <Q1). Both results confirmed that the genes associated with the GWAS phenotypes are critical to the treatment response ( Supplementary Table S9). According to the volcano plot, 32 expression probes among the high-response group were upregulated, compared to 49 in the low-response group, while 36 probes were downregulated in the high-response group and 41 in the low-response group ( Figure 2F and Supplementary Tables S10,11). Interestingly, the probes with significant fold changes in the high-response group showed strong associations with the GH-IGF-1 axis, such as the Wnt signaling pathway, liver development, and insulin secretion, indicating that these probes potentially promote the GHD treatment response. To identify SNPs that could impact the therapeutic response by regulating the transcription of certain genes, eQTL analysis was conducted through MatrixeQTL. Five eQTL associations between our SNPs and expression probes were found to be significantly correlated (FDR<0.05) ( Table 2 and Figure 2G–K). Notably, SNP rs12882504 of *SOS2* and probe 237640_at of *VCPKMT* are relatively close in position on the chromosome, and VCPKMT is a methyltransferase that trimethylates VCP Lys-315. As reported, the level of VCP methylation is positively correlated with the growth response in GHD patients [10] and with a marked reduction in growth development in VCPKMT-deficient cells, indicating that VCPKMT may affect the GHD response and that SOS2 may play a regulatory role.

**Table:** **Table 2** Significant eQTL associations between SNPs and expression probes included in the final prediction model

SNP (gene)	Expr-probe (gene)	FDR
rs12882504 ( *SOS2*)	237640_at ( *VCPKMT*)	1.84×10 ^‒12^
rs12908948 ( *IGF1R*)	213454_at ( *CENPS*)	2.02×10 ^‒3^
rs8017367 ( *SOS2*)	243531_at ( *LTO1*)	1.27×10 ^‒2^
rs13379306 ( *SOS2*)	243531_at ( *LTO1*)	1.57×10 ^‒2^
rs12718939 ( *EGFR*)	1552665_at ( *JMJD1C-AS1*)	1.74×10 ^‒2^

In summary, we successfully established a novel and promising model through the elastic net algorithm and identified significant biomarkers for the prediction of 1-month ΔIGF-1 in GHD children following r-hGH therapy, which can aid in clinical judgement for medical professionals. Nevertheless, larger sample sizes and more complicated methods are needed to select variables to predict treatment response. Moreover, the current study applied candidate SNPs for analysis. Further fully utilizing genome-wide data may enable a more thorough investigation of the influencing factors for complex traits, which could aid in the discovery of potential targets. Our predictive model comprised 1217 probe sets and 2 covariates (BMI and baseline IGF-1). The included probes are strongly associated with body height phenotype and can effectively differentiate GHD patients at different response levels, which provides further insights for related studies. Additionally, the incorporation of GWAS phenotype enrichment modelling into the analysis could explain the findings from additional perspectives. Interestingly, the SOS2-VCPKMT pathway was found to have a significant cis-eQTL association, and a set of differentially expressed probes with greater absolute fold changes were found to be strongly correlated with the GH-IGF-1 axis, which provides potential targets for further research on GHD treatment response.

## Supporting information

24184supplementary_materials

## Supplementary Data

Supplementary Data is available at *Acta Biochimica et Biophysica Sinica* online.
