# Supplementary material for: Significant biomarkers for predicting 1-month changes in IGF-1 in growth hormone-deficient children following r-hGH therapy: Biomarkers predicting changes in IGF-1 in GHD children after r-hGH therapy
Source: Acta Biochim Biophys Sin (Shanghai). 2024 Jun 6;56(11):1706–10. doi: 10.3724/abbs.2024089 (PMC11733495; doi:10.3724/abbs.2024089)
Supplement: 24184supplementary_materials [file 24184supplementary_materials.pdf]

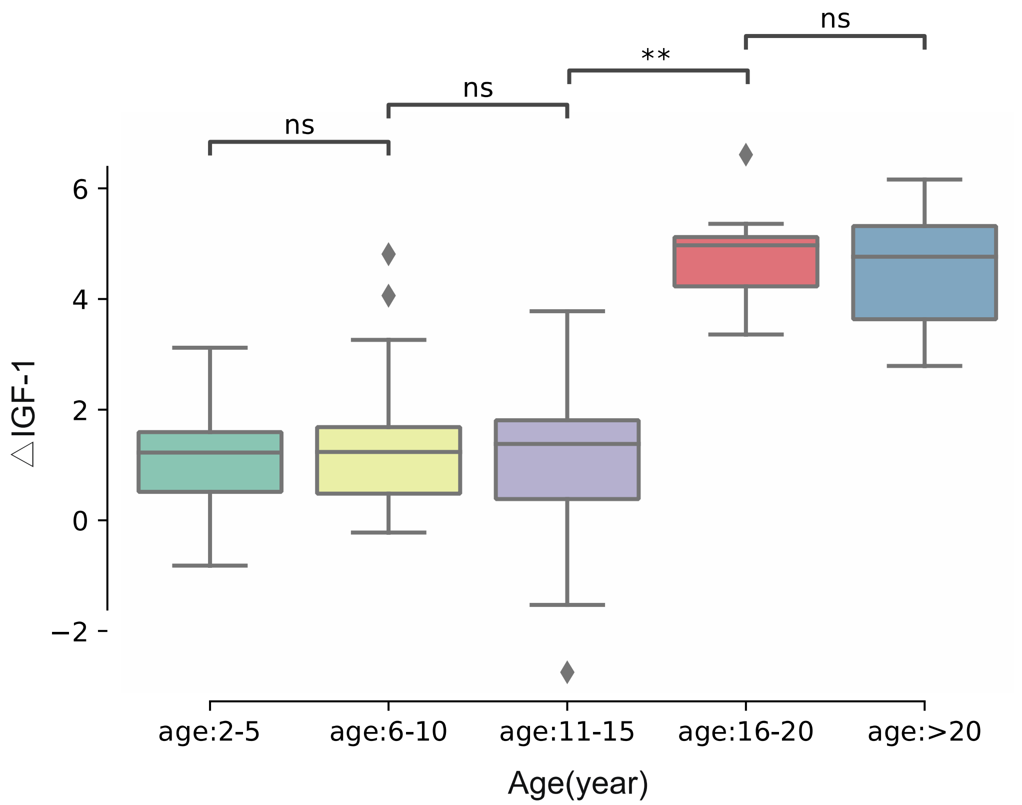

Supplementary Figure S1. Distribution of ΔIGF-1 in different age groups We grouped subjects into different age categories and made a boxplot for the ΔIGF-1 distribution within each age category. The ΔIGF-1 distribution of puberty subjects is completely different from that of the pre-puberty subjects, which suggests completely different factors such as hormone-driven changes impacting the treatment response. ns: not significant; ***P* <0.01.

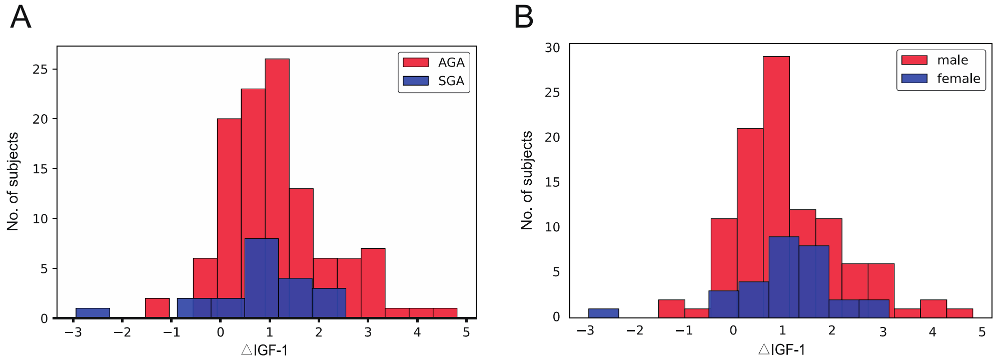

Supplementary Figure S2. Distribution of ΔIGF-1 in different gender and gestational age Kruskal Wallis test showed insignificance in both categories (Kruskal Wallis test *P* = 0.50 in different gender and Kruskal Wallis test *P* = 0.63 in different gestational ages).

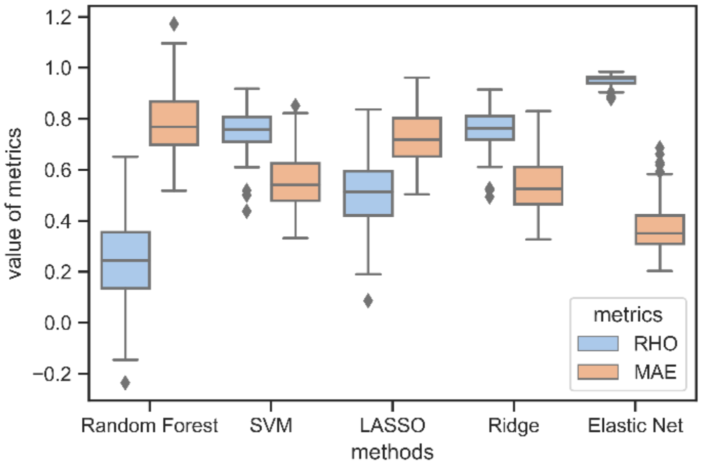

Supplementary Figure S3. Comparison of five machine learning methods when screening predictive factors Elastic net showed the highest performance with the lowest MAE, which is a multi-variable regularized regression model proved to be able to prune irrelevant variables for drug-response-related associations.

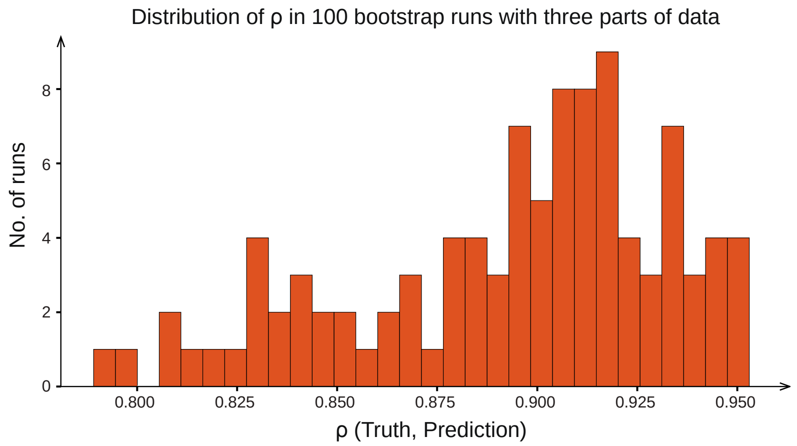

Supplementary Figure S4. The prediction model comprising three parts of data (expression probes/SNPs/clinical measurements) has a lower ρ (Spearman's rank correlation) than comprising only expression data and clinical measurements

Supplementary Table S1. The effect size and *P* value of the four individually significant clinical variables in multivariate regression to ΔIGF-1

Clinical marker	Coefficient	*P* value
BMI SDS	0.299	0.002
Baseline IGF-1 SDS	–0.303	0.009
Baseline IGFBP3 SDS	0.108	0.345
Baseline GH Peak	–0.009	0.798

Supplementary Table S2. Biological processes associated with 1536 SNPs used in this study

Biological process	*P* value
Insulin receptor signaling pathway	1.6×10^{-23}
Positive regulation of cell proliferation	3.3×10^{-20}
Cellular response to insulin stimulus	3.4×10^{-17}
Phosphatidylinositol-mediated signaling	2.1×10^{-16}
Negative regulation of apoptotic process	2.1×10^{-16}
Positive regulation of transcription from RNA polymerase II promoter	7.6×10^{-16}
Glucose homeostasis	2.5×10^{-15}
Phosphatidylinositol-3-phosphate biosynthetic process	2.9×10^{-15}
Glucose metabolic process	5.1×10^{-15}
Signal transduction	2.7×10^{-14}
Regulation of phosphatidylinositol 3-kinase signaling	4.2×10^{-14}
Ras protein signal transduction	2.9×10^{-13}
ERBB2 signaling pathway	2.9×10^{-13}
Phosphatidylinositol phosphorylation	5.1×10^{-13}
Epidermal growth factor receptor signaling pathway	6.0×10^{-13}

Supplementary Table S3. Single nucleotide polymorphisms (SNPs) in genes associated with growth response (ΔIGF-1) in GHD

Chr	Gene	SNP	*P* value
1	*LOC101929935*	rs11206883	0.0156
1	*HSD3B1*	rs6428830	0.0250
		rs1047303	0.0074
3	*RARB*	rs4681063	0.0383
		rs1286764	0.0407
3	*ADIPOQ*	rs1063537	0.0164
		rs1063539	0.0429
4	*PPARGC1A*	rs16874265	0.0458
		rs725289	0.0467
		rs2970872	0.0214
5	*PIK3R1*	rs12652661	0.0446
7	*GRB10*	rs2715107	0.0335
		rs6976572	0.0100
7	*EGFR*	rs13247687	0.0396
7	*CDK6*	rs2285332	0.0387
		rs2106135	0.0430
		rs17164721	0.0496
		rs2282989	0.0268
		rs3731283	0.0477
		rs3731267	0.0380
11	*CCND1*	rs649392	0.0217
11	*ARRB1*	rs567807	0.0255
		rs7929974	0.0269
15	*MIR7973-1*	rs3751592	0.0393
15	*IGF1R*	rs2684792	0.0386
17	*SLC2A4*	rs5435	0.0394
17	*PRKCA*	rs3803821	0.0476
18	*BCL2*	rs720321	0.0490
		rs2850763	0.0419
		rs2850762	0.0147
		rs1977971	0.0199
		rs4941195	0.0192
19	*INSR*	rs891087	0.0036
		rs6510960	0.0210
		rs10417205	0.0084
		rs919275	0.0103
		rs7254487	0.0133
20	*PTPN1*	rs734589	0.0351

Supplementary Table S4. Gene ontology biological process enrichment for significant SNPs

Biological process enrichment pathway	Genes	*P* value
Insulin receptor signaling pathway	*PTPN1*, *INSR*, *GRB10*, *PIK3R1*, *IGF1R*	5.66×10^{-7}
Positive regulation of protein phosphorylation	*CCND1*, *INSR*, *ADIPOQ*, *ARRB1*, *EGFR*	3.99×10^{-6}
Positive regulation of cell migration	*INSR*, *PRKCA*, *PIK3R1*, *EGFR*, *IGF1R*	1.73×10^{-5}
Cellular response to insulin stimulus	*INSR*, *ADIPOQ*, *SLC2A4*, *PIK3R1*	4.05×10^{-5}
Insulin-like growth factor receptor signaling pathway	*GRB10*, *PIK3R1*, *IGF1R*	6.74×10^{-5}
Positive regulation of glucose import	*INSR*, *ADIPOQ*, *PIK3R1*	3.19×10^{-4}
Brown fat cell differentiation	*ADIPOQ*, *SLC2A4*, *PPARGC1A*	3.64×10^{-4}
Negative regulation of apoptotic process	*BCL2*, *RARB*, *PIK3R1*, *EGFR*, *IGF1R*	5.73×10^{-4}
Positive regulation of cell proliferation	*INSR*, *BCL2*, *RARB*, *EGFR*, *IGF1R*	6.27×10^{-4}
Positive regulation of DNA replication	*INSR*, *EGFR*, *IGF1R*	6.28×10^{-4}
Negative regulation of ERK1 and ERK2 cascade	*PTPN1*, *ADIPOQ*, *ARRB1*	1.20×10^{-3}
Circadian rhythm	*ADIPOQ*, *PPARGC1A*, *EGFR*	1.99×10^{-3}
Signal transduction	*GRB10*, *RARB*, *ARRB1*, *PIK3R1*, *EGFR*, *IGF1R*	2.62×10^{-3}
Response to UV-A	*CCND1*, *EGFR*	2.68×10^{-3}
Transmembrane receptor protein tyrosine kinase signaling pathway	*INSR*, *EGFR*, *IGF1R*	3.24×10^{-3}
Cellular response to hypoxia	*BCL2*, *SLC2A4*, *PPARGC1A*	3.24×10^{-3}
Glucose homeostasis	*INSR*, *ADIPOQ*, *SLC2A4*	3.57×10^{-3}

Supplementary Table S5. Single nucleotide polymorphisms (SNPs) that are significantly associated with growth response (ΔIGF-1) in GHD

Chr	Gene	Related function or pathways	SNP	*P* value
1	*HSD3B1*	Enhance androgen receptor activation and promote cancer development	rs1047303	0.00741
			rs6976572	0.00996
19	*INSR*	Regulates glucose uptake and release, and associated with insulin resistance, involved in the MAPK/ERK and PI3K/AKT pathways	rs891087	0.00363
			rs10417205	0.00840

Supplementary Table S6. Top 50 predictive probes with the highest absolute effect sizes

Probe	Effect size	Probe	Effect size
1558053_s_at	-0.0095	220063_at	0.0072
210301_at	0.0093	233483_at	0.0071
202796_at	0.0087	213866_at	0.0071
232733_s_at	-0.0087	220050_at	-0.0071
232296_s_at	0.0085	1558533_at	0.0070
204011_at	-0.0084	208106_x_at	0.0070
220422_at	-0.0084	221240_s_at	0.0070
233103_at	-0.0083	218365_s_at	0.0070
1557311_at	-0.0082	205609_at	-0.0070
221987_s_at	0.0080	227952_at	-0.0070
216366_x_at	-0.0080	1557895_at	0.0069
237077_at	-0.0080	1570651_at	0.0069
1559050_at	-0.0079	224508_at	0.0069
240125_at	0.0076	233110_s_at	0.0069
244193_at	-0.0076	233909_at	0.0069
222270_at	0.0075	243660_at	0.0069
1568887_at	-0.0075	36084_at	0.0069
210955_at	-0.0075	201562_s_at	-0.0069
1568640_at	-0.0074	206632_s_at	-0.0069
202015_x_at	-0.0074	239637_at	-0.0069
217035_at	-0.0074	242237_at	-0.0069
204330_s_at	0.0073	232948_at	0.0068
1552498_at	-0.0073	213109_at	0.0068
1553181_at	-0.0073	236858_s_at	0.0068
209938_at	-0.0073	210161_at	-0.0068

Supplementary Table S7. Top 15 GWAS phenotypes associated with the genes included in the prediction model

GWAS phenotype	No. of genes	*P* value
Body height	129	0.03784
Blood protein measurement	97	0.00000
Heel bone mineral density	69	0.00235
Erythrocyte count	65	0.00002
BMI-adjusted waist circumference	64	0.00402
Eosinophil count	63	0.00004
Leukocyte count	58	0.00019
Waist-hip ratio	58	0.04162
Platelet count	57	0.00040
BMI-adjusted waist-hip ratio	54	0.04243
Mean corpuscular hemoglobin	54	0.00247
Mean corpuscular volume	54	0.04499
BMI-adjusted hip circumference	53	0.04176
High density lipoprotein cholesterol measurement	50	0.02736
Red blood cell distribution width	49	0.00629

Supplementary Table S8. KEGG pathways of 129 genes associated with GWAS phenotype "body height"

KEGG pathway	*P* value
Pathways in cancer	3.05×10^{-4}
HTLV-I infection	3.24×10^{-4}
Prostate cancer	3.39×10^{-4}
Signaling pathways regulating pluripotency of stem cells	3.88×10^{-4}
MAPK signaling pathway	1.70×10^{-3}
Proteoglycans in cancer	2.48×10^{-3}
Regulation of actin cytoskeleton	3.17×10^{-3}
Hepatitis B	3.21×10^{-3}
HIF-1 signaling pathway	4.33×10^{-3}
Ras signaling pathway	4.55×10^{-3}
Colorectal cancer	9.04×10^{-3}
PI3K-Akt signaling pathway	9.36×10^{-3}
Central carbon metabolism in cancer	9.86×10^{-3}
Renal cell carcinoma	1.07×10^{-2}
Ubiquitin mediated proteolysis	1.49×10^{-2}
Rap1 signaling pathway	1.49×10^{-2}

Supplementary Table S9. GWAS phenotype enrichment for 4705 expression probes whose expression changes were significantly associated with ΔIGF-1

GWAS phenotype	Count	*P* value
Body height	565	2.49×10^{-18}
Body mass index	319	4.64×10^{-06}
Blood protein measurement	289	2.79×10^{-06}
Self-reported educational attainment	279	5.69×10^{-06}
Protein measurement	252	1.11×10^{-01}
Heel bone mineral density	243	2.42×10^{-07}
Mean corpuscular volume	236	3.00×10^{-11}
Systolic blood pressure	215	7.74×10^{-03}
Mathematical ability	213	2.94×10^{-06}
Waist-hip ratio	212	2.05×10^{-04}
Erythrocyte count	211	4.08×10^{-10}
Mean corpuscular hemoglobin	206	6.53×10^{-11}
Intelligence	201	2.02×10^{-04}
Type II diabetes mellitus	199	2.30×10^{-04}
BMI-adjusted waist circumference	199	4.40×10^{-03}
Eosinophil count	198	3.57×10^{-08}
Leukocyte count	193	1.06×10^{-08}
FEV/FEC ratio	193	2.48×10^{-04}
BMI-adjusted waist-hip ratio	188	3.02×10^{-03}
Schizophrenia	188	1.23×10^{-02}
Platelet count	185	7.41×10^{-07}
BMI-adjusted hip circumference	185	2.49×10^{-03}
High density lipoprotein cholesterol measurement	174	7.78×10^{-04}
Red blood cell distribution width	168	2.16×10^{-05}

Supplementary Table S10. The number of up-/down-regulated expression probes in high-response and low-response groups and their enriched biological processes

Response level	The number of up-regulated probes	The number of down-regulated probes	Enriched biological processes ($P<0.01$)
High response	32	36	Regulation of transcription from RNA polymerase II promoter<br>Negative regulation of Wnt signaling pathway<br>Liver development<br>Pancreas development<br>Insulin secretion<br>Cell-matrix adhesion<br>Positive regulation of transcription, DNA-templated
Low response	49	41	Intracellular receptor signaling pathway<br>Positive regulation of cell migration

Supplementary Table S11. Up-/down-regulated expression probes in high-response and low-response groups

Response level	Up-regulated probes	Down-regulated probes
High response	223949_at	229095_s_at
	40093_at	217447_at
	1563943_at	1561719_at
	241625_at	207751_at
	215809_at	217204_at
	204044_at	1555365_x_at
	1568826_at	213276_at
	206566_at	1555011_at
	227058_at	207022_s_at
	236048_at	1560161_at
	221100_at	243639_at
	216975_x_at	240365_at
	215534_at	1554927_at
	224999_at	213493_at
	220518_at	210550_s_at
	221108_at	1554666_at
	1552779_a_at	207696_at
	209645_s_at	229430_at
	237621_at	230577_at
	209663_s_at	215199_at
	1565729_at	220886_at
	242360_at	244782_at
	235709_at	215849_x_at
	1570397_x_at	242034_at
	230308_at	1553243_at
	1569303_s_at	230920_at
	216930_at	212392_s_at
	226097_at	231031_at
	214324_at	204464_s_at
	214067_at	230087_at
	232863_at	206640_x_at
	243698_at	230611_at
		227417_at
		236096_at
		228427_at
		238308_at
Low response	215953_at	231892_at
	1557682_a_at	1566465_at
	202898_at	217276_x_at
	1556024_at	1563466_at
	243213_at	1564467_at
	1564790_at	1559650_at
	204315_s_at	237280_at
	228462_at	1552933_at
	1555116_s_at	216604_s_at
	244800_x_at	237020_at
	1553559_at	230567_at
	1564679_at	232085_at
	235069_at	202075_s_at
	243432_at	238340_at
	204259_at	219803_at
	214575_s_at	207778_at
	238710_at	1556366_s_at
	223908_at	223786_at
	1557874_at	209120_at

204830_x_at	208560_at
218416_s_at	243825_at
241122_s_at	232202_at
1552544_at	232458_at
227753_at	227628_at
233785_at	240212_at
215680_at	220594_at
211167_s_at	229158_at
202340_x_at	1554252_a_at
1563610_at	221305_s_at
224268_x_at	216915_s_at
1555294_a_at	206692_at
210498_at	216829_at
230754_at	240144_at
209714_s_at	234905_at
220779_at	205724_at
233574_at	1557080_s_at
234308_at	218663_at
234620_at	236638_at
1555798_at	243550_at
210906_x_at	211557_x_at
220106_at	220043_s_at
230554_at	
220733_at	
207444_at	
243793_at	
207569_at	
242087_x_at	
223868_s_at	
207546_at	
